# Barrier-Free Carrier Injection in 2D WSe_2_-MoSe_2_ Heterostructures via Fermi-Level Depinning

**DOI:** 10.3390/nano15131035

**Published:** 2025-07-03

**Authors:** Tian-Jun Dai, Xiang Xiao, Zhong-Yuan Fan, Zi-Yan Zhang, Yi Zhou, Yong-Chi Xu, Jian Sun, Xue-Fei Liu

**Affiliations:** 1 School of Electronic Information Engineering, Guiyang University, Guiyang 550005, China; 2 School of Physics and Electronic Science, Guizhou Normal University, Guiyang 550025, China; 3 School of Integrated Circuit, Guizhou Normal University, Guiyang 550025, China

**Keywords:** WSe_2_-MoSe_2_ heterostructure, Fermi-level pinning, Schottky barrier height, buffer layer, carrier injection efficiency

## Abstract

Fermi-level pinning (FLP) at metal–semiconductor interfaces remains a key obstacle to achieving low-resistance contacts in two-dimensional (2D) transition metal dichalcogenide (TMDC)-based heterostructures. Here, we present a first-principles study of Schottky barrier formation in WSe_2_-MoSe_2_ van der Waals heterostructures interfaced with four representative metals (Ag, Al, Au, and Pt). It was found that all metal–WSe_2_/MoSe_2_ direct contacts induce pronounced metal-induced gap states (MIGSs), leading to significant FLP inside the WSe_2_/MoSe_2_ band gaps and elevated Schottky barrier heights (SBHs) greater than 0.31 eV. By introducing a 2D metal-doped metallic (mWSe/mMoSe) layer between WSe_2_/MoSe_2_ and the metal electrodes, the MIGSs can be effectively suppressed, resulting in nearly negligible SBHs for both electrons and holes, with even an SBH of 0 eV observed in the Ag-AgMoSe-MoSe_2_ contact, thereby enabling quasi-Ohmic contact behavior. Our results offer a universal and practical strategy to mitigate FLP and achieve high-performance TMDC-based electronic devices with ultralow contact resistance.

## 1. Introduction

Among various two-dimensional (2D) materials, transition metal dichalcogenides (TMDCs) are attracting tremendous interest due to their promising applications in electronics [1,2], valleytronics [3], optoelectronics [4,5], and spintronics [6]. Specifically, the dangling bond-free surfaces of 2D TMDCs enable the flexible construction of van der Waals (vdWs) heterostructures, providing opportunities for new devices to be developed with advanced functions. Observing moiré superlattice exciton states in WSe_2_-WS_2_ vdWs heterostructures experimentally provides an attractive platform for controlling excited states of matter [7]. Tunneling field effect transistors based on 2D TMDC heterostructures, with a subthreshold swing of less than 60 mV dec^−1^, have been previously demonstrated [8,9]. Intriguingly, type II heterostructures can be constructed by employing appropriate TMDC layers, which enable the ultrafast dynamics of charge transfer across the vdWs interface and allow for the spatial segregation of photo-generated holes and electrons to be achieved. With 99% charge transfer efficiency, electrons can be transferred from WSe_2_ to MoS_2_ within 470 fs in MoS_2_-WSe_2_ p-n heterojunctions [10]. Policht et al. reported an ultrafast interlayer electron transfer time of up to 69 femtoseconds in a WS_2_-MoS_2_ heterostructure with type II band alignment, as revealed by two-dimensional electronic spectroscopy [11]. Similar sub-ps level charge transfer processes were also demonstrated in MoS_2_-MoTe_2_, MoSe_2_-MoS_2_ and WSe_2_-WS_2_ heterostructures [12,13]. However, when these heterojunctions are integrated into optoelectronic devices, the photoresponse times increase to the microsecond scale [14,15].

Inevitably, 2D TMDC-based devices have contact interfaces with metal electrodes, and the quality of the electrical contacts becomes particularly important for their performance. Unfavorable band edges are usually presented in 2D TMDCs due to the strong excitonic effect and quantum confinement [16]; thus, for most available metals, the work function varies from 3.5 eV to 5.7 eV [17]. The formation of low-resistance Ohmic contacts that do not obscure the intrinsic exceptional properties of two-dimensional (2D) TMDCs remains a significant challenge [18]. The Schottky barrier height (SBH) at metal–2D TMDC interfaces, influenced by complex Fermi-level pinning (FLP), does not solely depend on the difference between the metal work function and the valence band maximum (VBM) or conduction band minimum (CBM) of the TMDC. This presents a significant obstacle to efficient charge injection. A large SBH leads to high contact resistance at the metal–TMDC interfaces, reducing the carrier injection efficiency [19,20]. Therefore, it is highly desirable to decrease SBH to gain high performance for a device.

A Schottky pinning factor S is normally introduced to describe the strength of the FLP, which is defined as the change in SBH (Φ_SBH_) with respect to the metal work function (W_F_), i.e., S = |dΦ_SBH_/dW_F_| [17,21,22]. Note that S is the slope of Φ_SBH_ versus W_F_, typical values reported are in the range of 0~1 [17,21,22,23], where a value close to 0 corresponds to a strong pinning interface, and a weakly interacting metal–TMDC system is achieved for S close to 1, indicating the ideal Schottky–Mott limit. The FLP effects can normally be attributed to the interfacial effects [24], surface traps [25], and metal-induced gap states (MIGSs) [24,26,27]. Several studies have reported that the FLP can be weakened by inserting a buffer layer between the TMDCs and metal experimentally and theoretically, such as hexagonal boron nitride (h-BN) [28,29], molybdenum trioxide (MoOx, x < 3) [30], ultrathin TiO_2_ [31], graphene [32,33], ZnO [34], monolayer (ML) NbS_2_ [25], VS_2_ [26], and more. These results show that introducing buffer layers is an effective strategy to break the direct metal–TMDC interaction and eliminate the interface states, improving the contact properties.

In addition to their well-established advantages in ultrafast optoelectronics and emerging quantum information technologies [35,36,37], WSe_2_-MoSe_2_ heterostructures offer unique opportunities for fundamental studies in interface engineering. By using density functional theory (DFT) simulations, we investigate the interfaces between four conventional metals (Ag, Al, Au, and Pt) and the WSe_2_ and MoSe_2_ monolayers within the WSe_2_-MoSe_2_ heterostructure. Our results reveal universal Schottky-type contacts characterized by Fermi-level pinning. No ohmic contact is observed; MoSe_2_ forms an N-type Schottky contact with Ag and Al electrodes, exhibiting electron Schottky barrier heights (SBHs) of 0.31 eV and 0.88 eV, respectively. In contrast, p-type Schottky contacts are formed with Pt and Au electrodes, with hole SBHs of 0.42 and 0.76 eV, respectively. A similar trend is observed for metal–WSe_2_ interfaces, where the Pt-WSe_2_ contact exhibits the lowest hole SBH of 0.43 eV. Furthermore, we also demonstrated that inserting a metallic 2D interlayer of mMoSe or mWSe between the metal and the WSe_2_/MoSe_2_ layer significantly weakens the interaction at the contact interface. This effectively suppresses the MIGSs, reduces Fermi-level pinning, and substantially reduces all SBHs.

## 2. Computational Methods

The Projector Augmented Wave (PAW) method and plane-wave basis set implemented in the Vienna ab initio simulation package (VASP) code were employed to optimize the geometries [23,24]. The Perdew Burke Ernzerhof (PBE) exchange correction function under the generalized gradient approximation (GGA) function was utilized [38]. To ensure accuracy, the plane-wave cutoff energy was set as 600 eV. The Brillouin zone was sampled by special k-points of 5 × 5 × 1 for optimizing and 10 × 10 × 1 for densities of state (DOS) calculations [39,40]. The calculations were deemed converged when the energy difference between two successive steps was less than 10^−6^ eV, and the force acting on each atom is below 0.01 eV/Å. To account for van der Waals (vdW) interactions, we employed the DFT-D3 dispersion correction method developed by Grimme [41].

To accurately model metal electrode interfaces, six atomic layers of (111)-oriented Ag, Al, Au, and Pt slabs were employed, based on surface properties converging in previous studies [42,43]. The in-plane lattice constants of WSe_2_ and MoSe_2_ are a_1_ = 3.29 Å and a_2_ = 3.32 Å, respectively, demonstrating excellent agreement with experimental measurements [44,45]. To obtain stable heterojunction structures, a supercell with a lattice mismatch of less than 5% was required. Accordingly, pristine metal–WSe_2_/MoSe_2_ supercells were constructed using a 2 × 2 expansion of the (111) surfaces of Ag, Al, Au, and Pt, and a√3 × √3 expansion of monolayer (ML) WSe_2_/MoSe_2_. This resulted in lattice mismatches ranging from 0.21% to 3.56%, as summarized in Table 1. To eliminate spurious interactions between periodic images, a vacuum layer exceeding 15 Å in thickness was introduced along the z direction.

## 3. Results and Discussion

**Figure 1:**
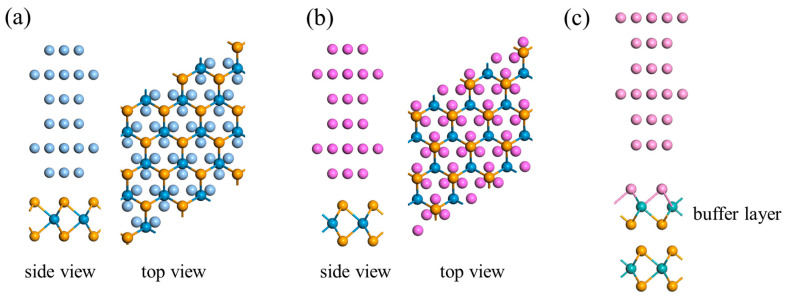
Interfacial geometries of contacts to ML WSe_2_/MoSe_2_. (**a**) Side and top views of Ag/Au (111)-WSe_2_/MoSe_2_ contacts. (**b**) Side and top views of WSe_2_/MoSe_2_ on Al/Pt (111) surfaces. (**c**) Side views of the metal–WSe_2_/MoSe_2_ contacts with a metal-doped mMoSe/mWSe buffer layer inserted.

Figure 1a,b illustrate the op-contact configuration architectures, in which monolayer WSe_2_/MoSe_2_ interfaces with six-layer (111)-oriented metal slabs of Al, Ag, Au and Pt. The most stable configuration of the ML WSe2/MoSe2 on Ag and Au corresponds to a geometry in which the Se atoms are located at the center of a hexagons formed by six adjacent surface metal atoms, directly above the surface hollow sites, while the W or Mo atoms are located above the center of a triangle formed by three neighboring metal atoms, as shown in Figure 1a. Figure 1b displays the most stable configuration of Al- and Pt-WSe_2_/MoSe_2_ interfaces, where the W or Mo atoms reside above the centers of surface hexagons, and the Se atoms lie above the centers of surface triangles formed by metal atoms. Since previous studies have demonstrated that structural and electronic properties show negligible variation beyond six layers of metal atoms [46], we restrict the slab thickness to six atomic layers. The equilibrium interfacial distances d_z_, defined as the vertical separation between the Se atoms and the topmost at the interface, range from 2.488 to 2.912 Å, decreasing in the order Al > Au > Ag > Pt (see Table 1). The binding energy per interfacial Se atom is defined as follows:

*E*_b_ = (*E*_WSe2/MoSe2_ + *E*_metal_ − *E*_metal-WSe2/MoSe2_)/*N_Se_* (1)

Here, *E*_metal_, *E*_WSe2/MoSe2_, and *E*_metal-WSe2/MoSe2_ represent the relaxed total energies of the isolated metal surface, WSe_2_/MoSe_2_, and the combined system per supercell, respectively; *N_Se_* denotes the number of Se atoms at the interface in each supercell. The calculated binding energy *E*_b_ ranges from 0.182 eV (Al-WSe_2_) to 0.342 eV (Pt-MoSe_2_), as summarized in Table 1, reflecting the trend in bonding strength. Notably, Pt exhibits relatively strong adsorption, with *E*_b_ values of 0.318 and 0.342 eV and interfacial distances of 2.53 and 2.488 Å for WSe_2_/MoSe_2_, respectively, indicating stronger bonding compared to the other metals.

**Figure 2:**
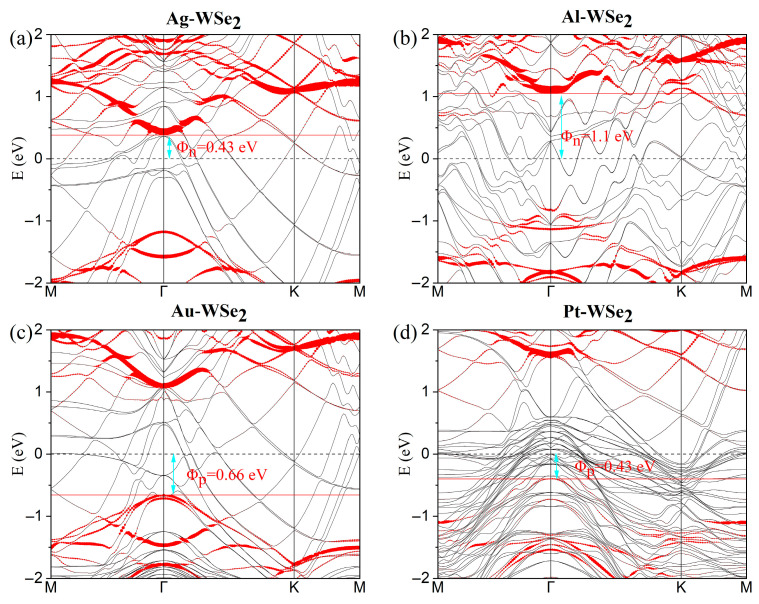
(**a**–**d**) Band structures of ML WSe_2_ in contact with several metals. The Fermi level is set to zero. The bands dominated by metal atoms and ML WSe_2_ are plotted using gray and red curves, respectively. The Schottky barrier is marked in red.

**Figure 3:**
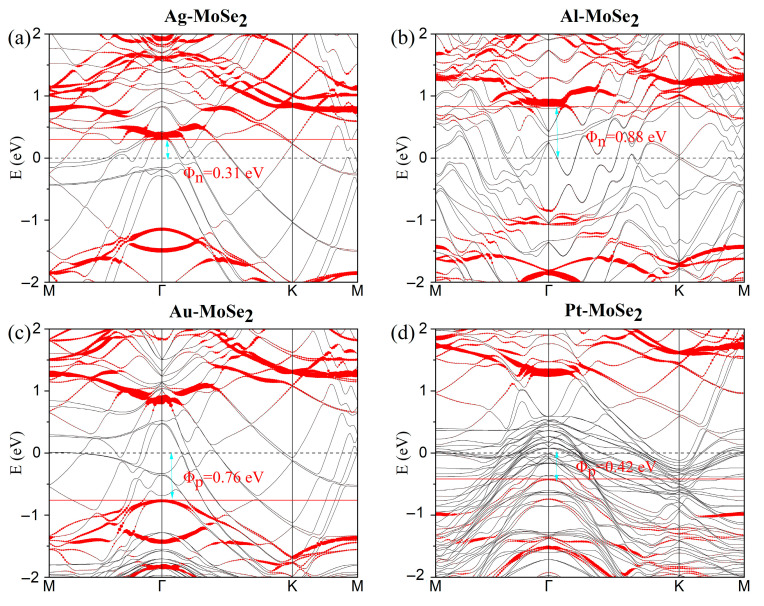
(**a**–**d**) Band structures of ML MoSe_2_ in contact with several metals. The Fermi level is set to zero. The bands dominated by metal atoms and ML MoSe_2_ are plotted using gray and red lines, respectively. The Schottky barrier is marked in red.

Prior to investigating the metal–TMDC interface systems, we calculated the band structures of pristine monolayer (ML) MoSe_2_ and WSe_2_ (see Figure S1 in the Supplementary Materials). The results confirm that ML WSe_2_/MoSe_2_ are semiconductors with band gaps of 1.63 and 1.51 eV, respectively, in good agreement with previously reported values [47,48]. Figure 2 presents the projected band structures of the metal–WSe_2_ systems, while those for metal–MoSe_2_ systems are shown in Figure 3. In all cases, the majority of the WSe_2_ and MoSe_2_ bands remain discernible upon contact with Ag, Al, Au, and Pt surfaces. Although the conduction bands of MoSe_2_/WSe_2_ are well preserved when interfaced with Pt, as shown in Figure 2d and Figure 3d, slight hybridization is observed in the valence bands. This hybridization is attributed to the radius and occupancy of the metal d-orbital [48]. Actually, the degree of band hybridization across the various metal–MoSe_2_/WSe_2_ interfaces can be interpreted using the d-band model [49]. Pt, possessing partially filled d-orbitals, forms stronger bonds with MoSe_2_/WSe_2_ than Au and Ag, which have fully filled d-shells. These d-orbitals can interact with the d-band edge states of W and Mo, thereby enhancing electron injection efficiency. This observation is consistent with our binding energy calculations. Conversely, Al lacks d-orbitals, resulting in weak bonding with TMDCs [43,47]; accordingly, high contact resistance in Al-TMDC systems has been experimentally verified [50].

Upon contact formation, Schottky barriers arise in the vertical direction. The vertical Schottky barrier height can be precisely determined using Φ_n_ = E_CBM_ − E_F_ for electrons and Φ_p_ = E_F_ − E_VBM_ for holes, where E_CBM_, E_VBM_, and E_F_ represent the conduction band minimum, valence band maximum, and Fermi level of the metal–TMDC junction, respectively. Φ_SBH_ plays a critical role in determining the optical and electronic properties of MoSe_2_/WSe_2_ device. As shown in Figure 2a,b, the Ag- and Al-WSe_2_ interfaces form n-type Schottky contacts, with electron SBHs of Φ_n_ = 0.43 and 1.1 eV, respectively. In contrast, the Au- and Pt-WSe_2_ contacts (see Figure 2c,d) exhibit p-type behavior, with hole SBHs of Φ_p_ = 0.66 eV and 0.43 eV. The extracted SBHs values for all metal–WSe_2_ systems are summarized in Table 1. By using the same approach, we evaluated the SBHs of the metal–MoSe_2_ systems, as shown in Figure 3, with results also compiled in Table 1. Specifically, the Ag- and Al-MoSe_2_ interfaces exhibit n-type SBHs of 0.31 eV and 0.88 eV, while the Au- and Pt-MoSe_2_ contacts exhibit p-type SBHs of 0.76 eV and 0.42 eV, respectively.

Notably, the large Φ_n_ value for Al-MoSe_2_/WSe_2_ indicates high resistance at the contact interfaces, which is consistent with the previous analysis. The contact polarity of Au-WSe_2_ has often been reported as ambipolar or p-type [51,52,53]. However, n-type contact behavior can be induced by selenium vacancies [24]. A significant reduction in p-type SBH has been reported for ML WSe_2_ [52], demonstrating that the SBH value can be tuned by different fabricated techniques. Furthermore, the vertical hole SBHs of Pt-MoSe_2_/WSe_2_ systems (0.42/0.43 eV) are comparable to previously reported values of 0.55/0.34 eV [47,48].

Contact engineering is essential for reducing the Φ_SBH_ of the metal–TMDC systems to achieve a low-resistance Ohmic contact in 2D devices. One technologically feasible strategy involves the introduction of an ultrathin buffer layer to lower Φ_SBH_. For instance, inserting a hexagonal boron nitride (hBN) buffer layer at a Ni-MoS_2_ interface has been shown to reduce the SBH from 0.158 to 0.031 eV [54]. Similarly, a graphene buffer layer decreases the SBH from 0.3 to 0.19 eV in an Ag-MoS_2_ configuration [33]. In addition, thin oxide layers such as Al_2_O_3_ have been used as a buffer layer at Ti-MoS_2_ interfaces, reducing the SBH from 0.18 to 0.13 eV [55]. In this study, we propose that metal-doped MoSe_2_/WSe_2_ (mMoSe/mWSe) can serve as an effective buffer layer when inserted between metal electrodes and semiconducting MoSe_2_/WSe_2_, as illustrated in Figure 1c. A naturally formed van der Waals (vdW) interface exists between the mMoSe/mWSe buffer and the underlying semiconductor layer. Notably, substitutional doping where metal atoms replace Se atoms requires less formation energy and is more thermodynamically favorable than substitution at Mo or W sites. Experimentally, an mMoS layer has been successfully synthesized via a three-step plasma deposition–annealing–yttrium doping process [56]. The transformation of semiconducting MoSe_2_/WSe_2_ into metallic mMoSe/mWSe by metal doping was theoretically predicted. As an example, Au doping results in a metallic band structure for AuMoSe_2_/AuWSe_2_, with the conduction band minimum (CBM) and valence band maximum (VBM) coinciding in the Brillouin zone, confirming the zero-band gap metallic nature (see Figure S2).

**Figure 4:**
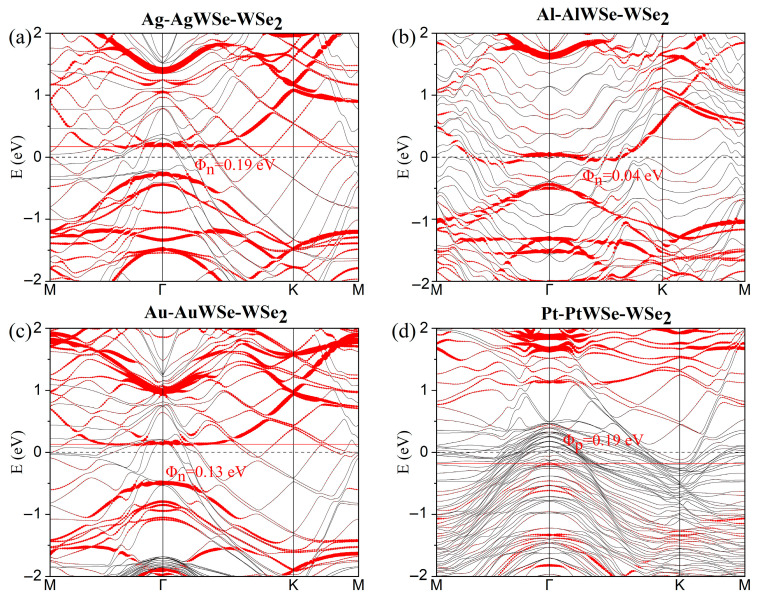
(**a**–**d**) Projected band structures of several metals in contact with ML WSe_2_ with inserted mWSe buffer layers. The Fermi level is set to zero. Red line: energy bands dominated by the WSe_2_ layer; gray line: band structures of metal–mWSe systems.

**Figure 5:**
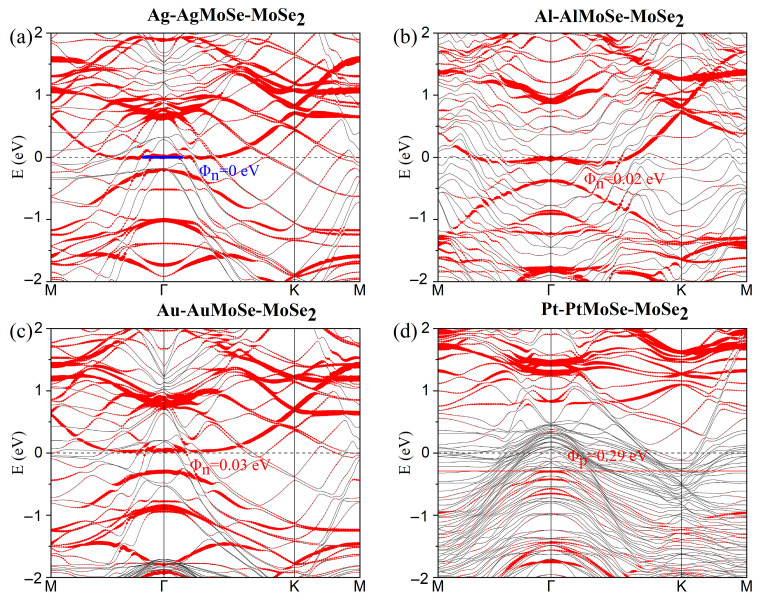
(**a**–**d**) Projected band structures of several metals in contact with ML MoSe_2_ with inserted mMoSe buffer layers. The Fermi level is set to zero. Red line: energy bands dominated by the MoSe_2_ layer, gray line: band structures of metal–mMoSe systems.

Figure 4a–d show the projected band structures of metal–mWSe–WSe_2_ heterostructures. The insertion of a 2D mWSe buffer layer at the contact interface reduces the Schottky barrier heights to 0.19, 0.04, 0.13, and 0.19 eV for the Ag-, Al-, Au-, and Pt-mWSe-WSe_2_ systems, respectively. Similarly, for metal–mMoSe–MoSe_2_ configurations (Figure 5a–d), the extracted vertical SBHs are 0, 0.02, 0.03, and 0.29 eV, respectively. All extracted electron and hole SBHs are summarized in parentheses in Table 1. It is evident that Φ_n_ in n-type contacts is significantly reduced, while Φ_p_ in p-type contacts shows only moderate reduction when compared with the corresponding systems without a buffer layer.

Interestingly, in contrast to previous conclusions, the ultralow SBHs observed for Al-mMoSe/mWSe-MoSe_2_/WSe_2_ systems demonstrate that Al can become a viable contact metal for low-resistance applications upon inserting a suitable buffer layer. Moreover, a contact polarity transition is observed: while Au-MoSe_2_/WSe_2_ interfaces typically exhibit p-type behavior, the corresponding Au-mMoSe/mWSe-MoSe_2_/WSe_2_ structures display n-type characteristics. Furthermore, the Ag-mMoSe-MoSe_2_ system exhibits a vanishing vertical n-type SBH (Figure 5a), indicating the formation of a low-resistance Ohmic or quasi-Ohmic contact, which implies enhanced carrier injection efficiency.

**Figure 6:**
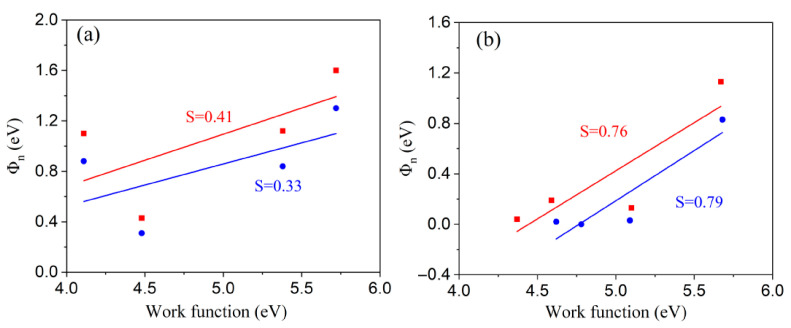
Variation in the Schottky barrier height of electrons (Φ_n_) with change in the work function for metal systems (**a**) in metal–MoSe_2_/WSe_2_ stacks and (**b**) in metal–mMoSe/mWSe–MoSe_2_/WSe_2_ stacks. The red and blue solid lines are fitted curves for metal systems in contact with WSe_2_ and MoSe_2_, respectively. Fermi-level depinning is seen after inserting a mMoSe/mWSe buffer layer, with pinning factors increased significantly.

The extracted Schottky barrier heights Φ_SBH_ for various configurations deviate from the values predicted by straightforward band alignment estimations, which can be attributed to the effect of Fermi-level pinning (FLP) at the interfaces. To further quantify the strength of the pinning behavior in different contacts, we plot the dependence of Φ_n_ on the metal work function for MoSe_2_ and WSe_2_ semiconductor systems, as shown in Figure 6. It is observed that the SBHs for metal–WSe_2_ interfaces lie along a line with a slope of S = 0.41, while those for metal–MoSe_2_ interfaces follow a line with a slope of S = 0.33 (Figure 6a). These slopes deviate significantly from the ideal Schottky–Mott limit (S = 1), indicating a strong Fermi-level pinning effect. Notably, these values are considerably larger than those observed in conventional semiconductor systems such as GaAs (S = 0.07) and Si (S = 0.27) [57], highlighting the partial tunability of Fermi-level positioning in TMDCs.

For comparison, Figure 6b shows the extracted pinning factors when a mMoSe/mWSe buffer layer is inserted between the metals and the MoSe_2_/WSe_2_ semiconductors. The resulting pinning factors are significantly larger: 0.79 for metal–mMoSe–MoSe_2_ and 0.76 for metal–mWSe–WSe_2_, indicating a substantial depinning of the Fermi level. This depinning behavior is attributed to the reduced density of metal-induced gap states (MIGSs) in the contact region, arising from a weakened interaction between the metal electrodes and the MoSe_2_/WSe_2_ layers. Previous studies have also shown that increasing the metal–TMDC interlayer distance can effectively suppress MIGSs and thereby contribute to Fermi-level depinning and SBH reduction [23].

**Figure 7:**
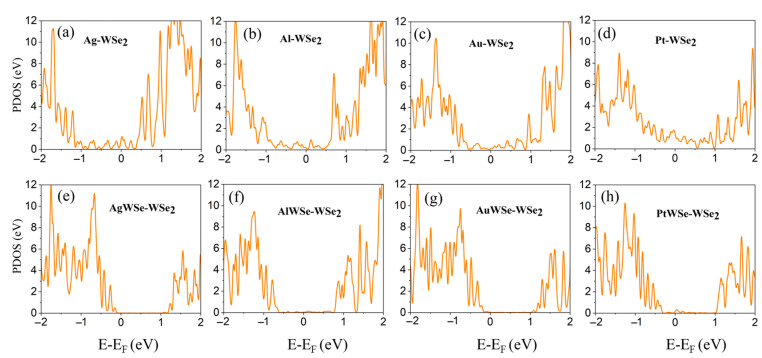
Partial density of states (PDOS) of WSe_2_ after contact with (**a**–**d**) metals and (**e**–**h**) metallic mWSe surfaces for comparison. The Fermi level is at zero energy.

To gain deeper insight into the effects of conventional metals and metal-doped layers (mMoSe/mWSe) on monolayer (ML) MoSe_2_ and WSe_2_ semiconductors, we calculated the partial density of states (PDOS) for both pristine MoSe_2_/WSe_2_ and their corresponding contact systems. As shown in Figure 7a–d, significant metal-induced gap states (MIGSs) appear within the intrinsic band gap of WSe_2_ upon contact with metal surfaces, in contrast to the pristine WSe_2_, which shows a clean band gap (see Figure S3). These findings are consistent with previous studies [17,47]. Among the investigated metal contacts, Pt-WSe_2_ exhibits the most pronounced MIGS distribution within the band gap (Figure 7d), indicating strong interfacial hybridization. This observation aligns well with the degree of band structure hybridization discussed earlier. In contrast, as shown in Figure 7e–h, when a metal-doped metallic mWSe buffer layer is introduced at the interface with ML WSe_2_, the resulting systems exhibit negligible MIGSs, leading to minimal Fermi-level pinning. This can be attributed to the formation of a native, nearly ideal van der Waals (vdW) interface between the mWSe and WSe_2_ layers. Moreover, a similar trend is observed in the MoSe_2_ systems. A comparison of the PDOS between metal–MoSe_2_ and mMoSe-MoSe_2_ configurations (see Figure S4 in the Supplementary Materials) reveals consistent behavior, further validating the role of the metallic buffer layer in suppressing interfacial states and reducing Fermi-level pinning.

**Figure 8:**
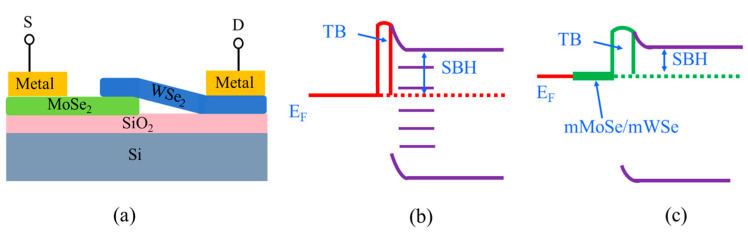
(**a**) Schematic diagram of a WSe_2_-MoSe_2_ heterostructure device. (**b**) Simplified band diagram for metal–MoSe_2_/WSe_2_ contacts, and the SBH and TB depend on the type of metal, which are related to the band hybridization degree. (**c**) Sketch of the band diagram for metal–mMoSe/mWSe-MoSe_2_/WSe_2_ stacks. The barrier height is reduced by suppressing the penetration of MIGSs.

A prototype electronic/optoelectronic device based on a WSe_2_-MoSe_2_ van der Waals (vdW) heterostructure, comprising a source (S), a drain (D), and a heterostructure channel, is illustrated in Figure 8a. The interfacial distance d_z_, obtained from geometry optimization (Table 1), and a notably high potential drop ΔV, defined as the potential energy above the Fermi level E_F_ at the metal–WSe_2_/MoSe_2_ interfaces, are observed (Figure S5), confirming the presence of tunneling barriers (TBs) [17,47].

In evaluating carrier injection efficiency, both tunneling barriers and Schottky barriers are considered. Taking the n-type contact as an example, band alignment diagrams for metal–WSe_2_/MoSe_2_ and metal–interlayer–WSe_2_/MoSe_2_ stacks are presented in Figure 8b,c, respectively. It is important to emphasize that a narrow tunneling barrier combined with a low Schottky barrier at the metal–TMDC interface is beneficial for enhancing carrier injection efficiency [17,18,47]. In the absence of a buffer layer, a thin or even vanishing tunneling barrier forms at the metal–WSe_2_/MoSe_2_ interface, which correlates with the degree of band hybridization (Figure 8b). Although the insertion of an mMoSe or mWSe interlayer introduces a larger interfacial distance due to the non-bonding vdW gap between the interlayer and the monolayer MoSe_2_/WSe_2_, this configuration significantly weakens the interfacial interaction. As a result, the formation of metal-induced gap states (MIGSs) is effectively suppressed (Figure 8c), thereby reducing the Schottky barrier heights (SBHs).

## 4. Conclusions

The strategic modulation of Schottky barrier heights (SBHs) for both carrier polarities constitutes a critical advancement in developing high-efficiency two-dimensional optoelectronic devices. In this work, we systematically investigated the contact interfaces between the constituent monolayers of a WSe_2_-MoSe_2_ heterojunction and a set of metals with a wide range of work functions (Ag, Al, Au, and Pt). Our calculation results show that directly contacting WSe_2_/MoSe_2_ with various metals results in partial FLP due to the large density of MIGSs, and the electron/hole SBHs can be modulated effectively by varying metals (with relatively large values ranging from 0.31 eV for the Ag-MoSe_2_ contact to 1.11 eV for the Al-WSe_2_ contact). A larger value of S close to 1 can be obtained by inserting a metal-doped mWSe/mMoSe layer as a metallic buffer between the metals and the 2D WSe_2_/MoSe_2_ semiconductor, suggesting a depinning Fermi-level effect. In the presence of a buffer layer, both the polarities of Au-MoSe_2_ and Au-WSe_2_ contacts change from P-type to N-type, and all SBHs can be reduced to a small even negligible value due to the suppression of MIGSs, implying a low-resistance Ohmic contact. Our studies provide a theoretical reference for developing high-performance 2D WSe_2_-MoSe_2_ heterostructure devices. Although our study focuses on equilibrium electronic structures, advanced topics like light-induced Floquet states or plasmonic effects in 2D systems could further enrich the understanding of TMDC-based heterostructures and are worth exploring in future work.

## Figures and Tables

**Table 1:** Calculated interfacial properties of WSe_2_ and MoSe_2_ on the metal electrodes. LM represents the lattice constant of the metals used in this article, with lattice mismatches in parentheses below. d_Z_ is defined as the physical separation (the distance between the topmost metal atomic layer and the Se atoms in the z direction). E_b_ is the binding energy. W and W_M_ are the calculated work functions for metal–WSe_2_/MoSe_2_ contacts and clean metal, respectively. The SBHs are extracted from the band calculation without (with) the insertion of a buffer layer (^N^ electron Schottky barrier, and ^P^ hole Schottky barrier).

Metal	LM (Å)	WSe_2_/MoSe_2_				
		d_Z_ (Å)	E_b_ (eV)	W_M_ (eV)	W (eV)	SBH (eV)
Ag	5.778 (1.26/0.40%)	2.719/2.683	0.284/0.305	4.48	4.59/4.78	0.43 ^N^/0.31 ^N^ (0.19 ^N^/0.00 ^N^)
Al	5.726 (0.36/0.49%)	2.912/2.784	0.182/0.205	4.11	4.37/4.62	1.1 ^N^/0.88 ^N^ (0.04 ^N^/0.02 ^N^)
Au	5.767 (1.07/0.21%)	2.879/2.780	0.252/0.261	5.38	5.1/5.09	0.66 ^P^/0.76 ^P^ (0.13 ^N^/0.03 ^N^)
Pt	5.549 (2.73/3.56%)	2.53/2.488	0.318/0.342	5.72	5.67/5.68	0.43 ^P^/0.42 ^P^ (0.19 ^P^/0.29 ^P^)

## Data Availability

The original contributions presented in this study are included in the article/Supplementary Material. Further inquiries can be directed to the corresponding author(s).

## Supplementary Materials

The following supporting information can be downloaded at: https://www.mdpi.com/article/10.3390/nano15131035/s1.
