# Supplementary material for: Barrier-Free Carrier Injection in 2D WSe2-MoSe2 Heterostructures via Fermi-Level Depinning
Source: Nanomaterials (Basel). 2025 Jul 3;15(13):1035. doi: 10.3390/nano15131035 (PMC12250957; doi:10.3390/nano15131035)
Supplement: Supplementary file 1 [file nanomaterials-15-01035-s001.zip › nanomaterials-3709076-supplementary.pdf]

Barrier-Free Carrier Injection in 2D WSe_2-$MoSe_2$ Heterostructures via Fermi-Level Depinning

Tian-Jun Dai[1*], Xiang Xiao[1], Zhong-Yuan Fan[1], Zi-Yan Zhang[1], Yi Zhou[1], Yong-Chi Xu[1], Jian Sun[1], Xue-Fei Liu[2,3*]

[1]School of Electronic Information Engineering, Guiyang University, Guiyang 550005, China

[2]School of physics and electronic science, Guizhou Normal University, Guiyang, 550025, China

[3]School of Integrated Circuit, Guizhou Normal University, Guiyang, 550025, China.

*Corresponding authors.

E-mail: daitianjun7227@163.com

E-mail:201307129@gznu.edu.cn

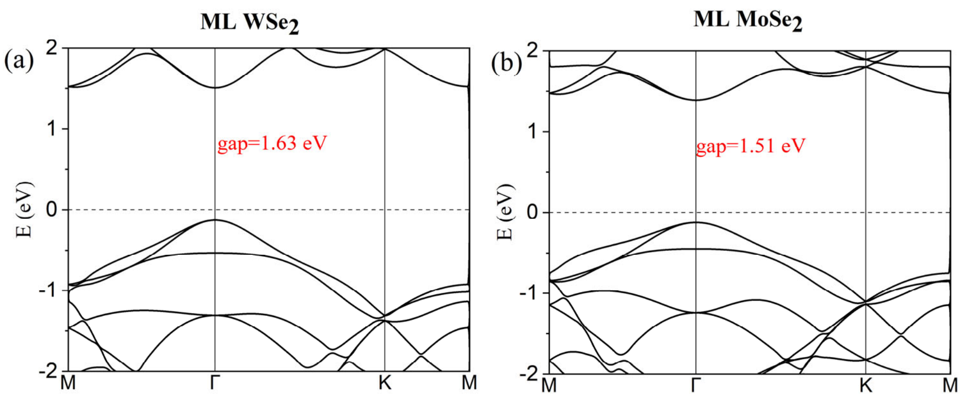

Figure S1. Band structures of (**a**) pure ML WSe_2($\sqrt{3}\times\sqrt{3}$) and (**b**) ML $MoSe_2$($\sqrt{3}\times\sqrt{3}$), exhibiting a direct band gap of 1.63 eV and 1.51eV, respectively.

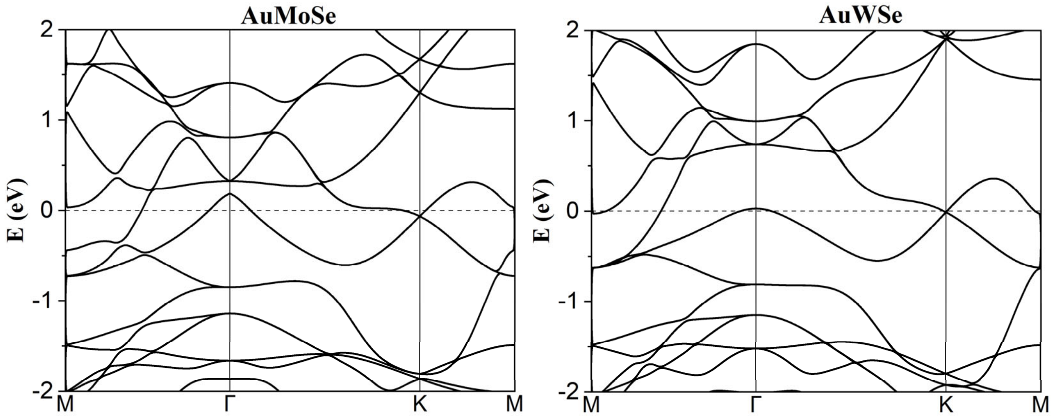

Figure S2. Band structures of Au-doped $MoSe_2$ and WSe_2, the zero-bandgap metallic characteristics were obtained.

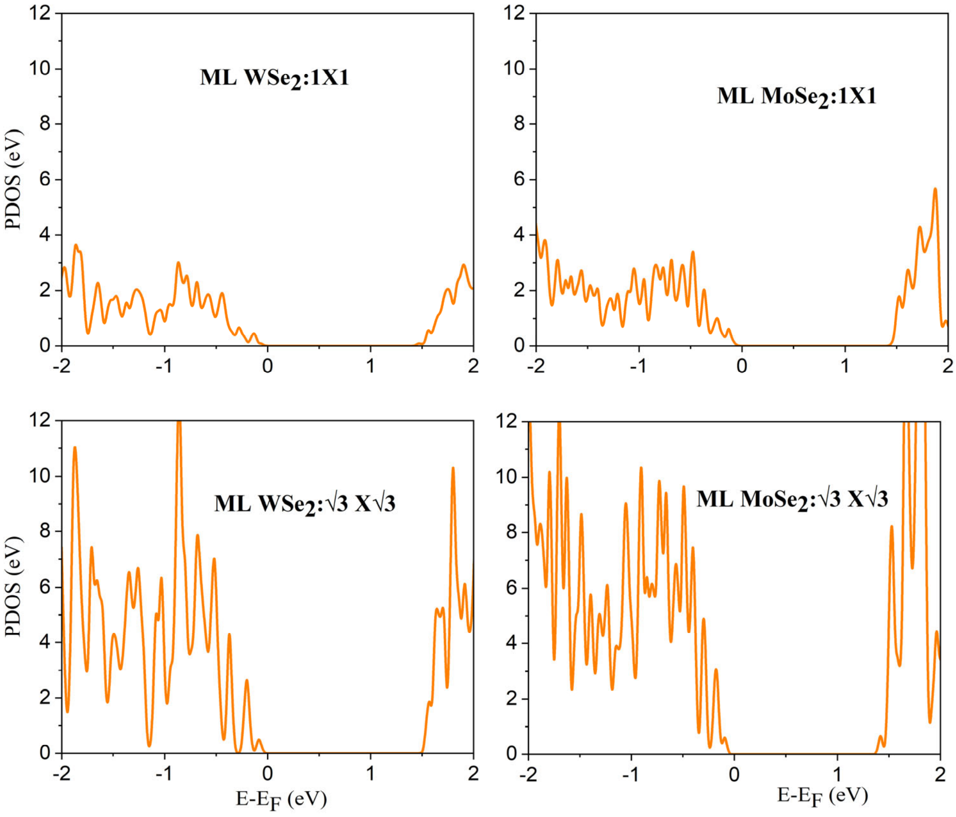

Figure S3. Partial density of states (PDOS) for pristine ML WSe_2 and $MoSe_2$. The Fermi level is set at zero.

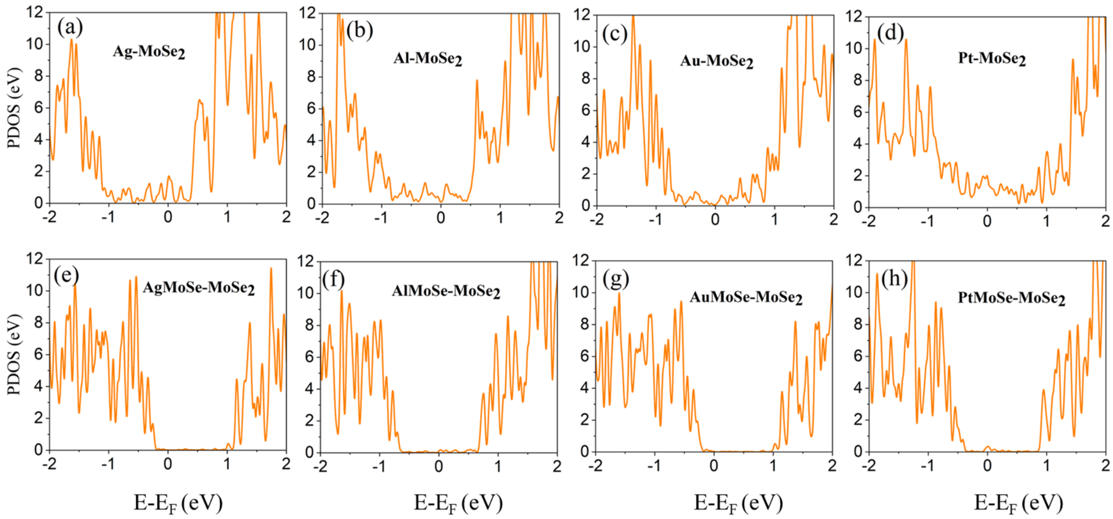

Figure S4. Partial density of states (PDOS) of $MoSe_2$ after contact with (**a-d**) metals and (**e-h**) metallic mMoSe surfaces. The Fermi level is at zero energy.

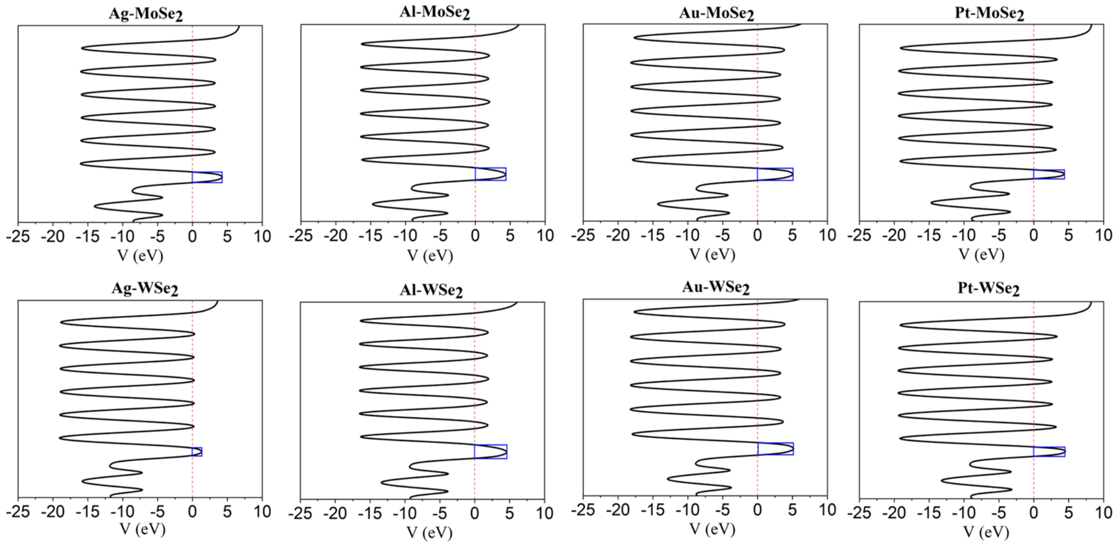

Figure S5. Average electrostatic potential V in planes normal to ML metal-WSe_2/$MoSe_2$ contacts. The Fermi level is zero, as indicated by the red dash lines.
